# Validation of less-invasive hemodynamic monitoring with Pulsioflex in critically ill patients: interim results of a multicentre study

**DOI:** 10.1186/cc12127

**Published:** 2013-03-19

**Authors:** K Van de Vijver, C Pigozzi, L Vervliet, V Vanbiervliet, V Brabers, I Vos, H Maes, N Van Regenmortel, I De laet, K Schoonheydt, H Dits, J Belda, Z Molnar, M Malbrain

**Affiliations:** 1 Ziekenhuis Netwerk Antwerpen, ZNA Stuivenberg, Antwerp, Belgium; 2 Hospital Clinico Universitario, Valencia, Spain; 3 University of Szeged, Hungary

## Introduction

Thermodilution (TD) is considered a gold standard for measurement of cardiac index (CI) in critically ill patients. The aim of this study is to compare intermittent bolus TD CI with intermittent automatic calibration CI (AutoCI) and two continuous CIs obtained by pulse contour analysis with PiCCO_2 _(PiCCI) and Pulsioflex (PuCCI).

## Methods

Interim results of an ongoing prospective multicentre study in 53 patients. Age 58.7 ± 15.4, SAPS II score 51.4 ± 14.7 and SOFA score 10 ± 3.2. All patients underwent PiCCO monitoring via a femoral line whilst a radial line was kept in place during four 8-hour time periods (in the first two periods, the Pulsioflex was connected to a radial line; in the last two it was connected to a femoral line). In the first and third periods, the Pulsioflex was calibrated with TDCI, for the second and fourth periods Pulsioflex was calibrated with AutoCI. Simultaneous PiCCI and PuCCI measurements were obtained every 2 hours while simultaneous TDCI and AutoCI were obtained every 8 hours. We also looked at the effects of 40 interventions.

## Results

In total, 940 CCI and 382 TDCI values were obtained: 940 paired PiCCI and PuCCI; 358 paired AutoCI-TDCI measurements. TDCI values ranged from 1.5 to 6.9 l/minute/m^2 ^(mean 3.6 ± 1.1), AutoCI from 1.8 to 7.2 (3.6 ± 0.9), PiCCI from 1.0 to 7.1 (3.5 ± 1.1) and PuCCI from 1.3 to 7.6 (3.6 ± 1). Pearson's correlation coefficient comparing mean PuCCI and PiCCI values per patient had an *R*^2 ^of 0.79. Comparison between AutoCI and TDCI had an *R*^2 ^of 0.51. Changes in AutoCI correlated well with changes in TDCI (*R*^2 ^= 0.44, concordance coefficient = 95.7), as did changes in PuCCI versus changes in PiCCI (*R*^2 ^= 0.99, CC = 93.4%). Changes in PiCCI and PuCCI induced by an intervention correlated well with each other (*R*^2 ^= 0.86, CC = 100%). The percentage error (PE) obtained by Bland and Altman analysis and *R*^2 ^for the different comparisons are presented in Table 1.

**Table 1:** Results of Bland and Altman analysis

Pulsioflex	AutoCal	PE (CCl) (%)	*n*	*R* ^2^	PE (TD-Pi) (%)	*n*	*R* ^2^	PE (TD-Pu) (%)	*n*	*R* ^2^
All	All	37.9	940	0.73	22.8	382	0.88	38.5	382	0.66
All	Yes	43.4	510	0.50	20.4	210	0.88	42.3	210	0.47
All	No	27.8	430	0.83	25.6	172	0.85	32.5	172	0.74
Fem	All	30.6	464	0.73	20.2	192	0.88	33.0	192	0.66
Rad	All	44.2	476	0.58	25.2	190	0.85	43.7	190	0.56

## Conclusion

The preliminary results indicate that in unstable critically ill patients, CI can be reliably monitored with Pulsioflex technology via a femoral line. Pulsioflex was also able to keep track of changes in CI.

